# New Insight into the Possible Roles of L-Carnitine in a Rat Model of Multiple Sclerosis

**DOI:** 10.3390/brainsci14010023

**Published:** 2023-12-25

**Authors:** Sally M. Safwat, Moutasem Salih Aboonq, Mahmoud El Tohamy, Moaz Mojaddidi, Saeed Awad M. Al-Qahtani, Madaniah Omar Zakari, Ahmed A. ElGendy, Abdelaziz M. Hussein

**Affiliations:** 1 Department of Medical Physiology, Faculty of Medicine, Mansoura University, Mansoura 35516, Egypt; sallysafwat186@gmail.com (S.M.S.); dr.m.eltohamy@gmail.com (M.E.T.); dr_ahmedelgendy@hotmail.com (A.A.E.); 2 Department of Medical Physiology, College of Medicine, Taibah University, KSA, Medina 42353, Saudi Arabia; aboonq@yahoo.co.uk (M.S.A.); mmojaddidi@taibahu.edu.sa (M.M.); dr_alqahtani@hotmail.com (S.A.M.A.-Q.); mzakari@taibahu.edu.sa (M.O.Z.)

**Keywords:** cuprizone, demyelinating, L-carnitine, Nrf2, neuroinflammation

## Abstract

Objective: We investigated the effect of L-carnitine (LC) on cuprizone (Cup) demyelinating rat model and its possible underlying mechanisms. Methods: Thirty male Sprague–Dawley (SD) rats were randomly allocated to three groups: the normal control group; the Cup group, in which Cup was administrated at a dose of 450 mg/kg per day orally via gastric gavage for 5 weeks; and the Cup + LC group, which received the same dose of Cup as the Cup group, except that the rats were treated additionally with LC 100 mg/kg/day orally for 5 weeks. The nerve conduction (NCV) in isolated sciatic nerves was measured; then, the sciatic nerves were isolated for H&E staining and electron microscope examination. The expression of myelin basic protein (MBP), IL-1β, p53, iNOS, and NF-KB by immunohistochemistry was detected in the isolated nerves. A PCR assay was also performed to detect the expression of antioxidant genes Nrf2 and HO-1. In addition, the level of IL-17 was measured by ELISA. Results: There was a significant reduction in NCV in the Cup group compared to normal rats (*p* < 0.001), which was significantly improved in the LC group (*p* < 0.001). EM and histopathological examination revealed significant demyelination and deterioration of the sciatic nerve fibers, with significant improvement in the LC group. The level of IL-17 as well as the expression of IL-1β, p53, iNOS, and NF-KB were significantly increased, with significant reduction expression of MBP in the sciatic nerves (*p* < 0.01), and LC treatment significantly improved the studied parameters (*p* < 0.01). Conclusion: The current study demonstrates a neuroprotective effect of LC in a Cup-induced demyelinating rat model. This effect might be due to its anti-inflammatory and antioxidant actions.

## 1. Introduction

The central nervous system is affected by the chronic, demyelinating, and inflammatory autoimmune illness known as multiple sclerosis (MS) [1,2]. There are around 2.5 million MS patients globally. Although the exact cause of MS is still unknown, environmental or genetic factors may play a role. Multiple brain areas can be affected by MS, including the striatum, corpus callosum, cortex, and white and gray matter. Multiple foci of inflammation, reactive gliosis (microgliosis and astrocytosis), oligodendrocyte depletion, demyelination, and axonal degeneration are all part of the pathological changes related to MS [3]. Experimental autoimmune encephalitis (EAE), Theiler’s murine encephalomyelitis virus, and toxin-induced demyelination (Cuprizone or Cup) are a few available animal models that have been reported to induce MS [4]. Myelin and axonal degradation and subsequent regeneration are two significant pathologies associated with MS that the Cuprizone (Cup) model can more accurately detect. For 5 to 6 weeks, a Cup dose can generate consistent demyelination; however, if this is continued for longer than 10 weeks, chronic demyelination is the usual result [5]. Recently, Mirzaie et al. [6] demonstrated a significant reduction in nerve conduction velocity (NCV) and in myelin sheath thickness in the sciatic nerves as peripheral nerves in a rat model of Cuprizone-induced neurotoxicity, suggesting the involvement of PNS in this process and confirm the involvement of myelin sheaths in PNS as well as CNS in multiple sclerosis.

Cytokines, particularly interleukin-17A (IL-17A or commonly known as IL-17), which is an inflammatory one, play critical roles in the inflammatory and neurodegenerative processes associated with MS [7]. Th-17 cells, as well as other CNS cells, including oligodendrocytes and astrocytes, release IL-17 [8]. Autoimmune illness is connected with high IL-17 expression. It binds to its IL-17R receptor, activating a variety of signaling cascades including nuclear factor kappa B (NF-KB) [9]. Additionally, IL-17 induces the synthesis of chemokines and the influx of neutrophils in microglia and astrocytes, which contribute to the onset or progression of MS [10]. It is important to remember that, although microglia is involved in clearing away dead cells and myelin debris, it also plays a role in the onset and progression of MS [11]. It has been demonstrated that microglia contain IL-17 receptors and that stimulation with IL-17A causes microglial activation and proliferation [8]. 

Additionally, prior investigations have shown that matrix metalloproteinases (MMPs), particularly MMP-9, have a role in the pathogenesis of MS [12]. Myelin basic protein (MBP), one of the most important elements of the myelin sheath, is destroyed as a result of MMP-9’s digestive impact [13]. Additionally, NF-ΚB is an inducible factor that is generated as a result of the activation of tumor necrosis factor (TNF) and interleukin-1 (IL-1) during the inflammatory process, resulting in the synthesis of pro-inflammatory mediators like MMP-9. As a result, one of the important inflammatory signaling pathways in microglia is NF-ΚB [2]. It is significant to note that MS produces cognitive dysfunction, mostly as a result of hippocampal demyelination, including reduced attention, slowed information processing, and long-term episodic memory [14]. On the other hand, inflammatory demyelinating illnesses have been linked to the pathophysiology of inducible nitric oxide synthase (iNOS). Peroxynitrite, a diffusible chemical that can harm membranes, cells, and nucleic acids, is produced when the highly poisonous molecule nitric oxide (NO), which is produced by the enzyme inflammasome (iNOS), combines with superoxide, which is created by inflammatory cells [15]. 

In addition, the tumor-suppressor gene p53 is a sequence-specific transcription factor with the capacity to cause cell-cycle arrest and apoptosis; this has been thoroughly studied. Members of the TNF receptor’s expression levels can be directly influenced by p53, which can result in immune-mediated damage in MS [16]. Furthermore, a number of studies point to oxidative damage as playing a significant role in the etiology of demyelination and neurodegeneration in multiple sclerosis (MS). This point of view is supported by the discovery of oxidized proteins, lipids, and DNA in MS lesions. The stimulation of the transcription factor nuclear factor (erythroid-derived 2)-like 2 (Nrf2), which, upon translocation into the nucleus, causes the production of a range of anti-oxidative defense molecules, is a well-known defense mechanism counteracting oxidative stress [17]. The gene heme oxygenase 1 (HO-1) is one of those controlled by Nrf2. The rate-limiting step in heme breakdown is catalyzed by this cytoprotective enzyme, which results in the production of equimolar quantities of iron ions, biliverdin, and carbon monoxide (CO). Important biological processes such as inflammation, death, cell proliferation, and fibrosis are regulated by HO-1 activity products [18].

L-carnitine (LC) is a necessary ingredient that is crucial for fatty acid metabolism and energy production. L-carnitine increases the efficiency of cellular energy generation by delivering long-chain fatty acids to the mitochondria for β oxidation during metabolism [19]. In various neuropathological contexts, according to several studies, LC can have neuroprotective benefits in Alzheimer’s disease [20], depression [21], Parkinson’s disease [22] and epilepsy [23]. Additionally, LC promotes cholinergic activation, protects against neurotoxicity, and improves cognitive function via its antioxidant and anti-inflammatory actions [19]. As a result, we propose that LC may be a promising and easily available neuroprotective drug for the treatment of demyelinating illnesses like multiple sclerosis. The goal of this study is to examine potential effects of LC on peripheral nerve structure and function a in a rat model of multiple sclerosis, as well as potential underlying mechanisms for such effects.

## 2. Materials and Methods

### 2.1. Experimental Animals

Thirty mature male Sprague Dawley rats weighing 150–200 g from Medical Experimental Research Center (MERC) Mansoura faculty of medicine, Mansoura University, were used in this study. In the animal house of MERC, animals were raised and housed. Rats were housed in cages with free access to food and water while being kept in environments with regulated humidity (40–70%), lighting (12 h cycle), and temperature (20–22 °C). The Mansoura Faculty of Medicine Committee on Animal Care and Ethics approved the experimental protocols and assigned it the code number R.22.06.1729 at 2 July 2022.

### 2.2. Experimental Design

Rats were divided into 3 groups, with 10 rats in each group:

1. Control group: Rats of this group were given 1.5 mL carboxymethylcellulose (CMC) purchased from Sigma Aldrich (St. Louis, MO, USA) daily by oral gavage for 5 weeks;
2. Cuprizone (Cup) group: Rats of this group were given 450 mg/kg of Cuprizone purchased from Sigma Aldrich (St. Louis, MO, USA) dissolved in 1.5 mL of 1% CMC per day orally for 5 weeks [5];
3. Cup + L-carnitine (LC) group: Rats of this group were given 450 mg/kg of Cuprizone dissolved in 1.5 mL of 1% CMC, per day orally + 100 mg/kg/day L carnitine purchased from El-Gomhoria Medical Company, Egypt orally for 5 weeks [19].

### 2.3. Animal Euthanasia and Collection of Samples

Rats were sacrificed on the final day of the experiment using thiopental anesthesia from Alpha chemical group company, Amreya, Egypt administered intraperitoneally at a dose of 30–40 mg/kg after being fasted the previous night. By creating a skin incision and severing the underlying muscles in the right and left thighs, the sciatic nerve of each rat was made visible. A left sciatic segment was fixed in 10% formalin from Alpha chemical group company, Egypt, for histological analysis. In order to conduct a quantitative real-time PCR analysis, another section of the left sciatic was kept frozen at −80 °C. The nerve conduction examination was conducted on the right sciatic nerve.

### 2.4. Nerve Conduction Study

Nerve conduction velocity (NCV) was measured by placing the isolated sciatic nerve specimen in a nerve chamber connected to a power lab 4/30 recording unit, and further analysis was conducted using lab chart 7 software (ADI instruments, Dunedin, Newzeland). A 37 °C ambient temperature and a pH of 7.4 were maintained with the help of Fresh Krebs solution [24]. Nerve conduction velocity (in m/s) was calculated by dividing the distance between the stimulating and recording electrodes (in meters) by the time interval between the stimulation and the start of the response (in seconds).

### 2.5. Histological Examination

Nerves (left sciatic nerves, 6 nerves from each group) were taken out for histological examination and immunohistochemical analyses. Samples of sciatic nerves were preserved in neutral formalin solution at 10%. Tissues that had been fixed in paraffin were serially cut into 5 µm slices. To assess the degree of neuronal injury and the state of demyelination, sciatic nerve slices were stained with of hematoxylin and eosin and Luxol fast blue (LFB) from Alpha chemical group company, Egypt, respectively [25]. The amount of demyelination in the sciatic nerve tissues was measured using ImageJ software (1.54h, NIH, Bethesda, MD, USA, 2023). (imagej.nih.gov), and the ROI was examined blindly by 2 operators.

### 2.6. Electron Microscopic Examination (TEM)

Freshly cut sciatic nerve slices from (4 nerves from each group) were fixed in 4% glutaraldehyde, and the tissue specimens were processed and analyzed [26]. The morphological changes in the sciatic nerves were described according to a previous study by Love, S. [27], who reported EM changes in a rat model of Cup-induced neurotoxicity. Also, the thickness of the myelin sheath in large-diameter fibers was calculated, as were the values of the g ratio (the ratio of the inner axon diameter to the outer diameter of the myelin sheath, which was used to assess axonal myelination for each group of nerve fibers) [28].

### 2.7. Evaluation of IL-17

The sciatic nerve samples (6 nerves from each group) that were stored at −80 °C were homogenized for the measurement of IL-17 using the ELISA technique with ELISA kits (Catalog# MBS175940, My Biosource, San Diego, CA, USA for IL-17).

### 2.8. Immunohistopathological Examination

Deparaffinization, rehydration, washing, submersion in 3% hydrogen peroxide, pepsin digestion, and antigen retrieval were all performed on the sciatic nerve sections. Sections were then cleaned with phosphate buffer. After the blocking of unspecific binding by serum, the sections were incubated with primary antibodies of myelin basic protein (Catalog # PA5-78397, Thermo Fisher Scientific, Waltham, MA, USA for MBP); IL-1β rabbit anti-rat IL-1β (Catalog# sc-7884, working dilution 1:100; Santa Cruz, CA, USA); iNOS (Catalog# iNOS (Catalog # 550339, BD Biosciences, San Diego, CA, USA; dilution 1: 100); NFκB (Santa Cruz, CA, USA, dilution 1: 150); and p53 (mouse monoclonal antibody, 1:100, ab90363, Abcam, Waltham, MA, USA). The number of positive cells and the degree of immunostaining in the region of interest (ROI) in 5 high-power fields (HPF) were calculated blindly by 2 operators using ImageJ (1.54h, NIH, Bethesda, MD, USA, 2023). (imagej.nih.gov) software.

### 2.9. PCR Assay of ANTIOXIDANT GENES (Nrf2/HO-1) 

RNAlater (10 μL per 1 mg of renal tissue) was employed to preserve samples of nerve tissue (catalog no. 76104, Qiagen, Hilden, Germany). The samples were then immersed in RNA and held for a further 24 h at 2–8 °C before being stored at −80 °C. Five strokes of liquid nitrogen were used to homogenize the tissue samples. The QIAzol reagent (Cat. No. 79306, Qiagen, Germany) was used to extract RNA. The purity and concentration of the resultant RNA were confirmed by Thermo-Scientific Nano Drop 2000 (Waltham, MA, USA). A Sensi FASTTM cDNA Synthesis Kit (Cat. No. 12594100) was used with an Applied Biosystems 2720 Thermal-Cycler (Applied Biosystems, Foster, CA, USA) to create the first strand of cDNA from 1 microgram of RNA.

Thermo-Scientific’s Pikoreal-96 real-time PCR machine was used to amp up the cDNA templates. The 20 μL total volume combination for the amplification reaction included 10 μL of HERA-SYBR green PCR Master Mix (Willowfort, UK), 2 μL of cDNA template, 2 μL of gene primer (10 pmol/μL), and 6 μl of nuclease-free water. The sequences of the primer pairs utilized for Nrf2 were [5′ATTGCTGTCCATCTCTGTCAG-3′.(sense) 5′-GCTATTTTCCATTCCCGAGTTAC-3′ (antisense)], and for HO-1, were [5′TGCTTGTTTCGCTCTATCTCC-3′.(sense) 5′-CTTTCAGAAGGGTCAGGTGTC-3′, (antisense)]. Glyceraldehyde-3-phosphate dehydrogenase (GAPDH) (5′-AGACAGCCGCATCTTCTTGT-3′, 5′-TTCCCATTCTCAGCCTTGAC-3′) was used as a reference gene. Using the Primer-BLAST program (https://www.ncbi.nlm.nih.gov/tools/primer-blast/, accessed on 19 December 2023), Primer specificity was confirmed (Vivantis, Selangor Darul Ehsan, Malaysia). Melting curve analysis was used to verify the specificity of the PCR products. The fold change of gene expression was calculated using the 2CT method, and relative gene expression levels were expressed as ΔCt = Ct target gene − Ct housekeeping gene; the 2^−ΔΔCT^ method was used to calculate the fold change of gene expression [28]. On 3% agarose gels, PCR products were run, and a UV transilluminator (OWI-Scientific, Antony, France) was used to visualize the results. The gels were then captured on camera with the BioRad gel documentation system (BioRad, Hercules, CA, USA).

### 2.10. Statistical Analysis

The data were expressed as mean ± SD. The data of the current study were normally distributed in the statistical analysis, so one-way (ANOVA) followed by Tukey’s post hoc test were used for measuring the statistical significance among all groups. *p* < 0.05 was considered significant.

## 3. Results

### 3.1. Effect of L-Carnitine (LC) on Cup-Induced Neurophysiologic Changes

**Figure 1:**
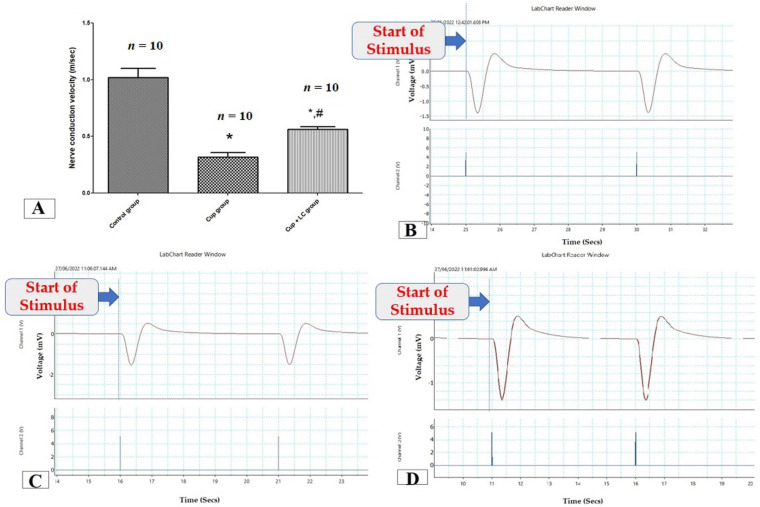
Nerve conduction study in isolated sciatic nerves. (**A**) Nerve conduction velocity (m/s) from different groups. Traces of NCS records from control group (**B**), Cuprizone group (**C**), and Cup + LC group (**D**). *: Significant vs. control group, #: significant vs. cuprizone group. *p* < 0.05 was considered significant.

To assess the function of the sciatic nerve, nerve conduction velocity was measured by a power lab. The NCV in isolated sciatic nerves in the Cup group was significantly lower than that in the control group (*p* < 0.001). On the other hand, it was significantly elevated in the LC group when compared with the Cup group (*p* < 0.001) (Figure 1A). Figure 1B,C are records of NCV from the control group, Cup group, and Cup + LC group, respectively. 

### 3.2. Effect LC on Sciatic Nerve Morphology 

**Figure 2:**
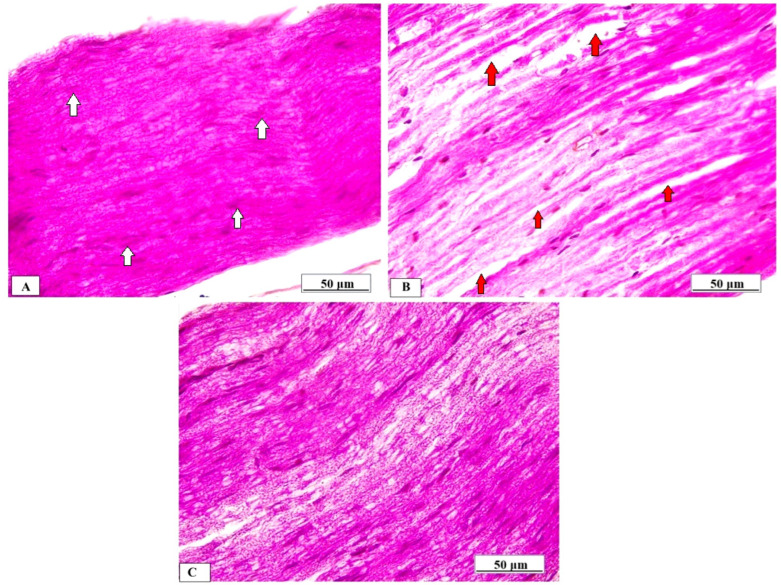
Histopathological examination of sciatic nerve by H&E from different studied groups. (**A**) Photomicrograph from normal control group shows dense, uniform, regular myelinated nerve fibers arranged as concentric layers with regular arrangement of Schwann cell nuclei (white arrows) (scale bar 50 μm). (**B**) Photomicrograph from Cup group shows myelinated fibers that were broadened, loose, and partially demyelinated (red arrows), with an irregular pattern of clear vacuoles, i.e., bubbling (red arrows) (scale bar: 50 μm). (**C**) Photomicrograph from Cup + LC group revealed regular myelin sheaths (white arrows) (scale bar: 50 μm).

Normal rats’ sciatic nerves displayed normal axonal histology, myelinated nerve fibers, and myelin sheaths that were uniformly packed and organized in concentric rings (Figure 2A). The myelinated fibers in the sciatic nerves of the Cup group, however, were widened, loose, and partially demyelinated, with an uneven pattern of distinct vacuoles, i.e., bubbling and inflammatory cell infiltrates (Figure 2B). On the other hand, the Cup + LC group revealed regular myelin sheaths similar to those of rats in the normal control group (Figure 2C).

**Figure 3:**
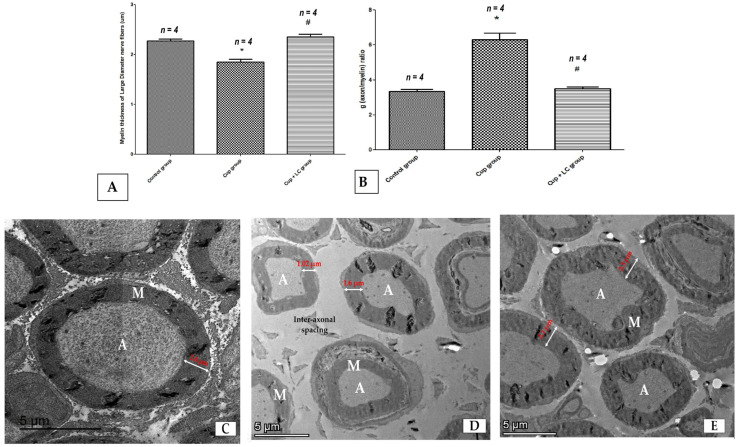
Ultra-microscopic structure of the sciatic nerve, shown by electron microscope images from different studied groups. (**A**) Score of myelin sheath’s thickness in micrometers in different groups; (**B**) g (axon/myelin) ratio in different groups; (**C**) photomicrograph from normal control group showing large myelinated nerve fibers with thick myelin sheaths (M) and axonal (A) cytoplasm with small-diameter nerve fiber axons surrounded by Schwann cells, ensheathing each axon in a pocket of its cytoplasm (scale bar 5 μm). (**D**) Photomicrograph from cuprizone group showing endoneurial edema (manifested by distinct displacement between nerve fibers and wide inter-axonal spacing (scale bar 5 μm); (**E**) photomicrograph from Cup + LC group showing intact large- and small-diameter axons near the normal control group (scale bar 5 μm). *: Significant vs. control group, #: significant vs. Cuprizone group. *p* < 0.05. A = axon, M = myelin.

Also, we used transmission electron microscopy to examine the ultrastructure of the sciatic nerve and the myelin sheath. The thickness of the myelin sheath of large-diameter nerve fibers showed a significant reduction in the Cup group compared to the normal control group (*p* < 0.001). The Cup + LC treated group showed a significant increase in myelin thickness in comparison with the Cup group (*p* < 0.001) (Figure 3A). Sciatic nerves from rats of the normal control group showed large myelinated nerve fibers with intact and thick myelin sheaths (M), as well as an axonal (A) cytoplasm containing mitochondria (m) with small-diameter nerve fiber axons surrounded by Schwann cells. These ensheathed each axon in a pocket of its cytoplasm, forming a Remak bundle. The normal thickness of the endoneurium implied a normal spacing of nerve fibers within the sciatic nerve (Figure 3B). On the other hand, the Cup group showed endoneurial edema (manifested by distinct displacement between nerve fibers), Wallerian degeneration (W) in axons of large-diameter fibers, and degeneration of non-myelinated nerve fibers surrounded by Schwan cells (Figure 3C). Moreover, the Cup + LC group exhibited preserved sciatic nerve structure with intact large- and small-diameter axons near the normal control group (Figure 3D).

### 3.3. Effect of LC on Cuprizone-Induced Demyelination

**Figure 4:**
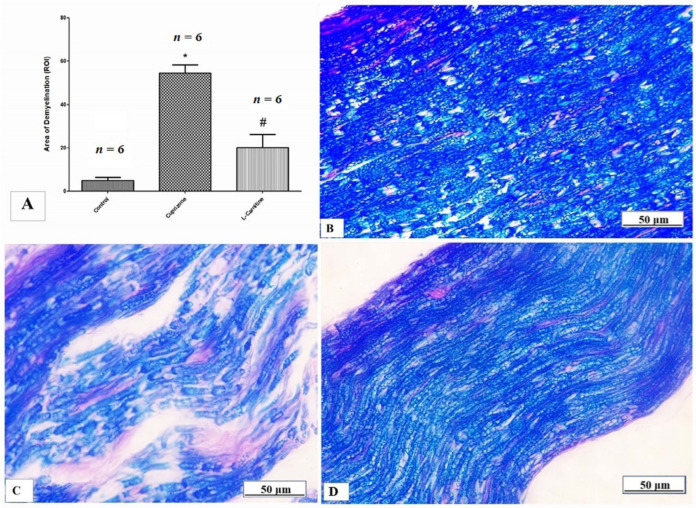
Luxol fast blue (LFB) staining for myelination of sciatic nerve. (**A**) The scores of demyelinating area in different studied groups. Photomicrographs of LFB staining from control group (**B**), cuprizone group (**C**), and Cup + LC group (**D**). *: Significant vs. control group, #: significant vs. Cuprizone group. *p* < 0.05 was considered significant.

LFB staining was used to determine the myelin status, and sciatic nerve immunostaining was used to analyze the MBP. When compared to the control group, the Cup group demonstrated severe demyelination, which was demonstrated by a substantial decrease in the intensity of LFB staining (*p* < 0.001). In contrast, the Cup + LC group showed a marked increase in the intensity of LFB staining (*p* < 0.001) (Figure 4A). Representative photomicrographs of LFB staining from the normal control group, Cup group, and Cup + LC group are shown in Figure 4B–D, respectively. 

**Figure 5:**
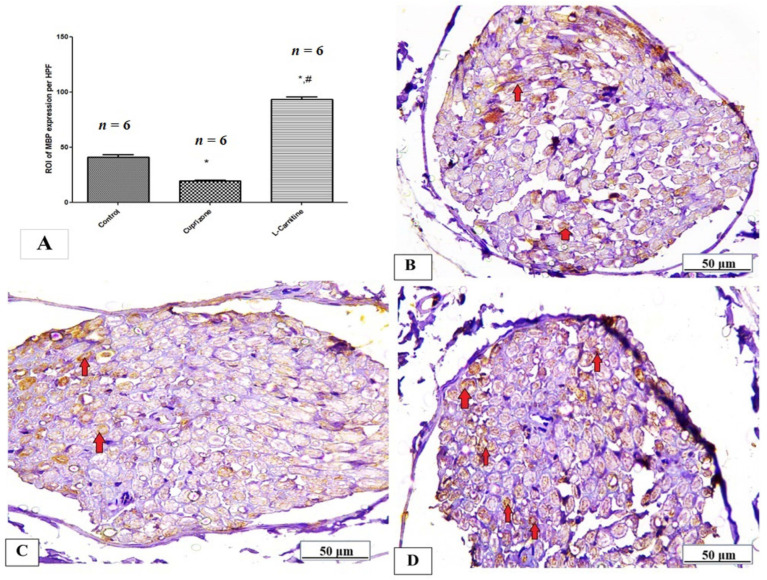
Immunohistopathological staining for myelin basic protein (MBP) in sciatic nerve. (**A**) the score of MBP expression in the region of interest (ROI) in different studied groups. Photomicrographs of MBP staining (brown membranous staining, red arrows) from control group (**B**), Cuprizone group (**C**), and Cup + LC group (**D**). *: Significant vs. control group, #: significant vs. Cuprizone group. *p* < 0.05 is considered significant.

Additionally, MBP significantly decreased in the Cup group compared to the regular group (*p* < 0.05). Compared to the Cup group, the Cup + LC-treated group showed a substantial increase in MBP expression in the sciatic nerve (*p* < 0.001) (Figure 5A). Figure 5B–D show representative photomicrographs of MBP immunostaining from the normal control group, Cup group, and Cup + LC group, respectively.

### 3.4. Effect of L-Carnitine on Cuprizone-Induced Neuroinflammatory Cytokines (IL-1β, iNOS, NF-κB, and IL-17) and Apoptotic Markers (p53)

**Figure 6:**
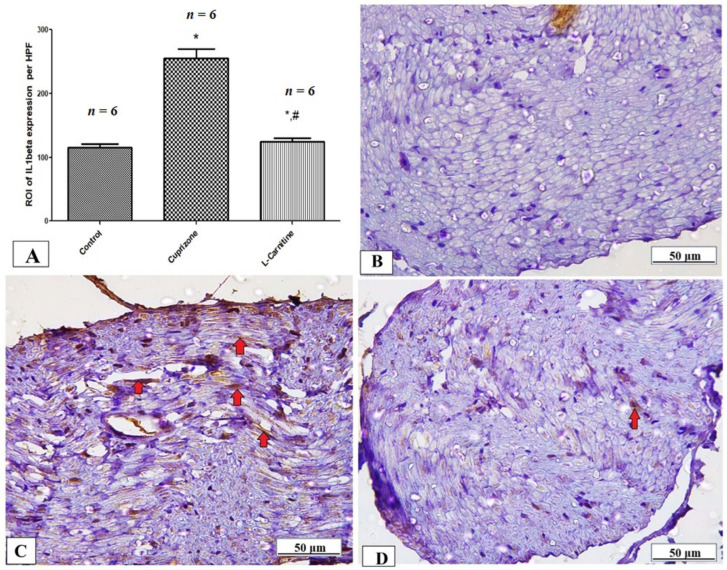
Immunohistopathological staining for IL1β in sciatic nerves. (**A**) The score of IL1β expression in the region of interest (ROI) in different studied groups. Photomicrographs of IL1β (brown cytoplasmic staining, red arrows) from control group (**B**), Cuprizone group (**C**), and Cup + LC group (**D**). *: Significant vs. control group, #: significant vs. Cuprizone group. *p* < 0.05 is considered significant.

**Figure 7:**
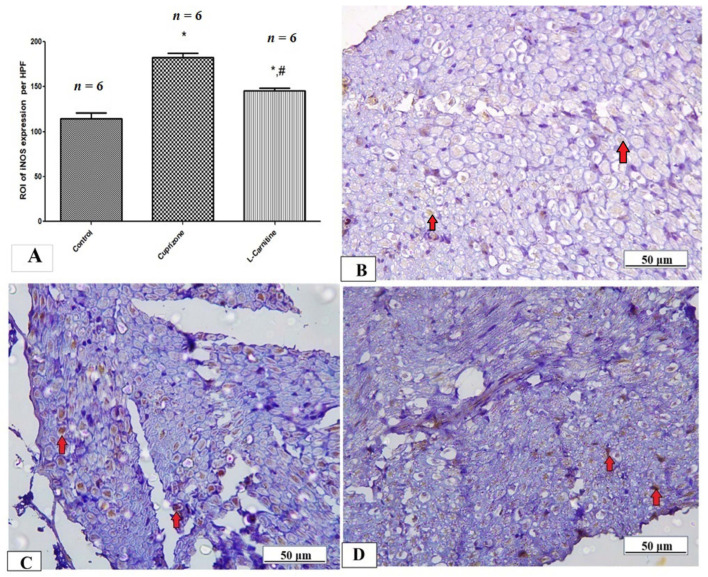
Immunohistopathological staining for iNOS in sciatic nerves. (**A**) The score of iNOS expression in the region of interest (ROI) in different studied groups. Photomicrographs of iNOS (brown cytoplasmic staining, red arrows) from control group (**B**), Cuprizone group (**C**), and Cup + LC group (**D**). *: Significant vs. control group, #: significant vs. Cuprizone group. *p* < 0.05 is considered significant.

**Figure 8:**
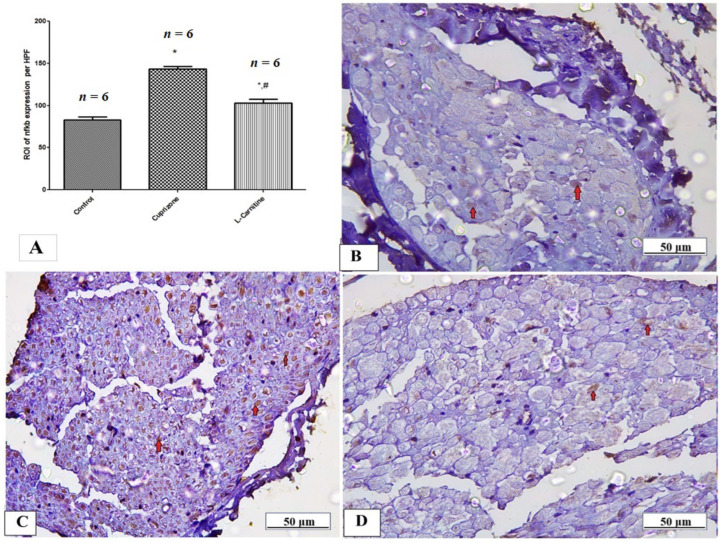
Immunohistopathological staining from NFκB in sciatic nerves. (**A**) The score of NFκB expression in the region of interest (ROI) in different studied groups. Photomicrographs of NFκB (brown nuclear staining, red arrows) from control group (**B**), Cuprizone group (**C**), and Cup + LC group (**D**). *: Significant vs. control group, #: significant vs. Cuprizone group. *p* < 0.05 is considered significant.

To assess neuro-inflammation, the expressions of IL-1β, iNOS, and NFκB at the protein level were analyzed by immunostaining in the sciatic nerve while the ELISA technique was used to assess the level of IL-17. The Cup group showed a significant increase in IL-1β, iNOS, NFκB, and IL-17 (*p* < 0.001) compared with the normal group. In contrast, the Cup + LC group showed significant reductions in IL-1β, iNOS, NFκB, and IL-17 (*p* < 0.001) compared with the Cup group (Table 1 and Figure 6A, Figure 7A and Figure 8A). Figure 6B–D, Figure 7B–D and Figure 8B–D show representative photomicrographs of IL-1β, iNOS, and NFκB immunostaining from the normal control group, Cup group, and Cup + LC group, respectively. 

**Figure 9:**
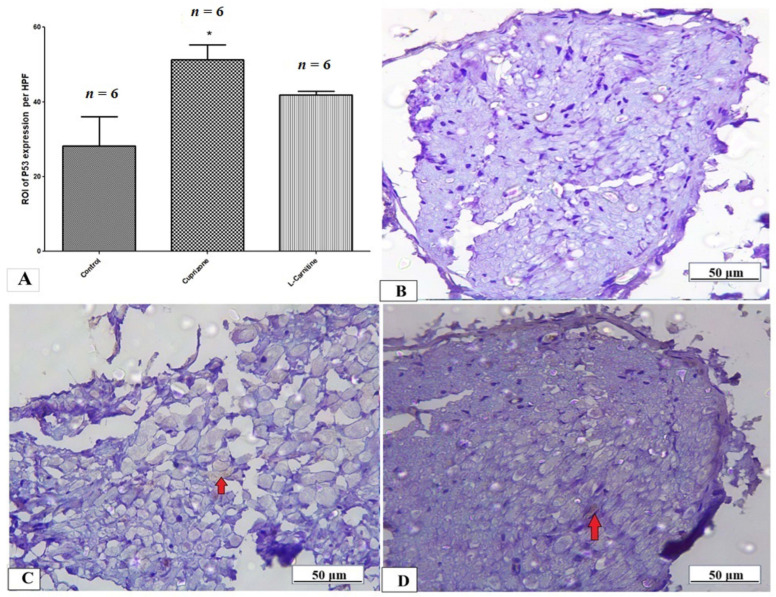
Immunohistopathological staining for p53 in sciatic nerves. (**A**) The score of p53 expression in the region of interest (ROI) in different studied groups. Photomicrographs of p53 from control group (**B**), Cuprizone group (**C**), and Cup + LC group (**D**). *: Significant vs. control group. *p* < 0.05 is considered significant.

The expression of the p53 protein in the sciatic nerve tissue was determined by immunostaining to provide information about the activation of the apoptotic pathway. Figure 9A shows a significant rise in the expression of the p53 protein in the Cup group compared to the normal control group, with no significant decrease in the Cup + LC group compared to the Cup group. Also, Figure 9B–D show representative photomicrographs of p53 immunostaining from the normal control group, Cup group, and Cup + LC group, respectively.

### 3.5. Effect of L-Carnitine on Expression of Antioxidant Genes (Nrf-2 and HO-1) at mRNA-Level Sciatic Nerve Tissues 

By using RT-PCR, we evaluated the mRNA levels of the antioxidant genes nuclear Nrf-2 (Nrf-2) and HO-1. The Cup group showed a significantly lower level of mRNA expression of Nrf-2 and HO-1 in the sciatic nerve (*p* < 0.05) when compared to control group. As opposed to the Cup group, the LC-treated group demonstrated a substantial increase in Nrf-2 and HO-1 (*p* < 0.001). Additionally, when the Cup + LC treated group was contrasted with the normal control group, there was a substantial elevation in both markers (*p* < 0.01) (Table 1).

## 4. Discussion

The main aim of this study was to analyze the possible role of L-carnitine (LC) in the Cuprizone (Cup)-induced demyelination of the sciatic nerves of rats. Multiple sclerosis (MS) is an autoimmune disease characterized by chronic inflammation, demyelination, and gliosis mainly affecting the central nervous system (CNS). However, peripheral nervous system (PNS) damage is also observed in traditionally perceived demyelination diseases [29].This also has been proven by electro-physiological as well as neuro-pathological examinations, which indicates the significant effect of MS on PNS [30].

The Cup-induced demyelination model, which closely matches the pathogenesis of MS in terms of myelin and axonal degeneration and subsequent regeneration, was used to achieve the goal of our study. A dose of Cup can cause chronic demyelination if taken for longer than 10 weeks; however, it can cause consistent demyelination for 5 to 6 weeks. Spontaneous remyelination occurs once Cup administration is ceased [5], so to assess the effect of LC on Cup induced demyelination, we administered it concomitantly with Cup from the start of our experimental period for 5 weeks. To support the occurrence of a peripheral demyelinating process, we investigated the alterations in the structure of the sciatic nerve by H&E, LFB, and MBP staining and transmission electron microscopy. Our study revealed that Cuprizone decreased LFB staining intensity and MBP expression compared with the control group, suggesting the development of demyelination of the PNS. Moreover, electron microscopic examination revealed significant deterioration of the sciatic nerve. Our result was in alignment with that of Yu et al. [31], who stated that Cuprizone induced neuronal pyknosis, degeneration, demyelination, and a significant reduction in the level of MBP in the hippocampus and cortex [31]. Moreover, the ultramicroscopic changes shown by EM in the sciatic nerve in the Cup group of the current study are in agreement with those described by Love [27].

On the other hand, treatment with LC markedly reversed the process of demyelination in the sciatic nerve and improved its microscopic structure and nerve conduction velocity, suggesting a neuroprotective role of LC against Cup-induced demyelination in PNS. Our results are in agreement with Gharighnia, Omidi [32], who showed that in the demyelinated mouse model, LC enhanced balance and motor coordination, suggesting that it may function as a therapeutic antioxidant during the myelin regeneration process.

A protective covering called myelin surrounds nerve fibers and aids in the conduction of action potentials in the neurological system. It participates in and speeds up the saltatory conduction of brain impulses. Schwann cells produce myelin in the peripheral nervous system, but oligodendrocytes are the myelinating cells in the central nervous system [33]. In the current study, we found demyelination in the sciatic nerve via LFB stain as well as downregulation of MBP in the sciatic nerves of the Cup group. The process of demyelination of nerve fibers was reflected by the conduction velocity of the nerve fibers, which was markedly reduced with demyelination. This is why we examined the conduction velocity in the current study on isolated sciatic nerve fibers. In the current research, we found a significant reduction in NCV in the Cup group compared to the control group, suggesting that the demyelination of nerve fibers resulted in a reduction in NCV in the sciatic nerves. This finding is in agreement with Ünsal and Özcan [34], who reported a significant reduction in sciatic nerve conduction velocity with Cup treatment in both male and female rats. Also, they reported that males were more sensitive to Cup than females, which is why we chose male rats in the current study. On the other hand, treatment with LC significantly attenuated the process of demyelination in the sciatic nerves, upregulated the expression of MBP, and enhanced the nerve conduction velocity, suggesting a remyelinating effect of LC against Cup-induced neurotoxicity in peripheral nerves. These findings are in line with those of Triana et al. [35], who reported that LC may have the function of stabilizing the process that leads to the integrity of myelin. Also, it has been shown that, following prolonged hypoperfusion in the rat brain, LC heals white-matter lesions and increases axonal plasticity [36].

The current study’s second objective was to investigate the potential mechanisms that might underlie Cup-induced neurotoxicity and LC’s protective effects in Cup-induced demyelinating rats. Cup, a copper-chelator, inhibits cytochrome oxidase and monoamine oxidase enzymes in mature oligodendrocytes, leading to mitochondrial dysfunction through enlargement or clustering, which is followed by their apoptosis, resulting in their death. The demyelination zones are populated by microglia and reactive astrocytes, which release pro-inflammatory cytokines such as TNF-α, interleukin-1, and interferon. Thus, the primary factors that control the Cup-induced demyelination process are neuroinflammation, oxidative stress, and apoptosis [37]. 

The process of neuroinflammation was the first mechanism we looked at, and we achieved this by measuring inflammatory cytokines in the sciatic nerve, including IL-1, IL-17, iNOS, and NF-KB. Important cellular processes are impacted by IL-1, including the reduction in DNA content, the reduction in protein synthesis and intracellular energy production, and the induction of cell death and necrosis [38]. Blood–brain barrier (BBB) endothelial cells secrete CCL2, IL-6, and IL-8 (CXCL8) in response to IL-17, which also stimulates the production of reactive oxygen species. It also stimulates the release of CXCL2 and IL-6 by microglia and of IL-6, IL-1, and nitric oxide (NO) by astrocytes, which heightens inflammation and attracts neutrophils [39]. The NF-kB/I-B pathway is triggered by NF-kB, which then enhances iNOS activity. The pathophysiology of intricate inflammatory illnesses is assumed to be significantly influenced by the overexpressed iNOS, which would lead to excessively elevated NO levels [40]. Our findings show that Cup significantly increased the levels of inflammatory mediators like IL-1, iNOS, NF-kB, and IL-17. This is in accordance with Abdel-Maged et al. [2]. These findings suggest the development of a neuroinflammatory process in the sciatic nerve with Cup feeding. Moreover, LC treatment significantly decreased these inflammatory markers in the sciatic nerves, which is in parallel with results of Koc et al. [41], who reported that nitric oxide generation, iNOS protein expression, and NF-kB activity were all reduced by LC. This anti-inflammatory action of LC has been reported in several previous studies [32,42].

Finally, there is compelling evidence that oxidative stress plays a role in MS. The increased ROS (oxidative stress) caused by Cup exposure causes or stimulates oligodendrocyte death [43]. Redox transcription factor Nrf-2 is thought to be a key regulator of HO-1 induction in the CNS, and is implicated in the activation of several antioxidants [44]. When compared to the normal group in the current investigation, nuclear Nrf-2 and HO-1 expression levels were considerably lower in the Cup group, and this had previously been reported by Abdel-Maged et al. [2]. However, LC increased the nuclear levels of Nrf-2 and HO-1 compared to the Cup group, which was in accordance with the results of Hota et al. [45], who revealed that LC-mediated neuroprotection during hypoxic insult occurs through Nrf-2-mediated regulation of mitochondrial biogenesis. Also, LC treatment was associated with increased levels of Nrf-2 and HO-1, which thought to be protective against retinal ganglion cell damage induced by high glucose levels as previously mentioned [46]. Lastly, it was demonstrated that oxidative stress and inflammatory process resulted in the upregulation of p53 and caspase-3 during the first 3 weeks of Cup administration [43]. In accordance with this finding, the current study demonstrated significant elevation in p53 expression in sciatic nerve tissues, suggesting the development of apoptosis in peripheral tissues with Cup intoxication. However, LC treatment to improve significantly the expression of p53 failed in the current work.

## 5. Conclusions 

In conclusion, LC established a reliable protective effect against Cup-induced demyelination in the peripheral nervous system and improved the structural and functional abnormalities in rat sciatic nerves. This neuroprotective effect is partially due to its anti-inflammatory effect of reducing IL-1β, IL-17, iNOS, and NF-KB, as well as its role in resisting oxidative stress via the upregulation of nuclear Nrf-2 and HO-1.

## Figures and Tables

**Table 1:** The concentration of IL17 (pg/mL) and the expression of antioxidant genes (Nrf2 and HO-1) in sciatic nerves of different groups.

	Control Group (*n* = 6)	Cuprizone Group (*n* = 6)	Cup + LC Group (*n* = 6)
Sciatic nerve IL17 (pg/mL)	47.50 ± 1.118	98.33 ± 2.789 ***	71.67 ± 4.014 ***^,##^
mRNA expression of Nrf2	0.98 ± 0.022	0.65 ± 0.014 *	1.65 ± 0.14 ***^,###^
mRNA expression of HO-1	1.000 ± 0.0056	0.65 ± 0.016 **	1.12 ± 0.098 ^###^

All data are expressed as mean ± SEM. One-way ANOVA with Tukey’s post hoc test. *: Significant vs. control group, ^#^: significant vs. Cuprizone group. * *p* < 0.05, ** *p* < 0.05, *** *p* < 0.001, ^##^ *p* < 0.01, ^###^ *p* < 0.001.

## Data Availability

The data presented in this study are available on request from the corresponding author. The data are not publicly available due to privacy.
